# Characterization of *Culex pipiens* Complex (Diptera: Culicidae) Populations in Colorado, USA Using Microsatellites

**DOI:** 10.1371/journal.pone.0047602

**Published:** 2012-10-19

**Authors:** Linda Kothera, Marvin S. Godsey, Michael S. Doyle, Harry M. Savage

**Affiliations:** Centers for Disease Control and Prevention, Division of Vector-Borne Diseases, Fort Collins, Colorado, United States of America; Instituto de Higiene e Medicina Tropical, Portugal

## Abstract

Mosquitoes such as those in the *Culex pipiens* complex are important vectors of disease. This study was conducted to genetically characterize *Cx. pipiens* complex populations in the state of Colorado, USA, and to determine the number of genetic clusters represented by the data. Thirteen populations located among four major river basins were sampled (n = 597 individuals) using a panel of 14 microsatellites. The lowest-elevation sites had the highest Expected Heterozygosity (H_E_) values (range 0.54–0.65). AMOVA results indicated the presence of statistically significant amounts of variation within each level when populations were analyzed as one group or when they were grouped either by river basin or by their position on the east or west side of the Rocky Mountains. Most pairwise F_ST_ values were significant via permutation test (range 0–0.10), with the highest values from comparisons with Lamar, in southeast CO. A neighbor joining tree based on Cavalli–Sforza and Edwards’s chord distances was consistent with the geographic locations of populations, as well as with the AMOVA results. There was a significant isolation by distance effect, and the cluster analysis resolved five groups. Individuals were also assayed with an additional microsatellite marker, Cxpq78, proposed to be monomorphic in *Cx. pipiens* but polymorphic in the closely related but biologically distinct species *Cx. quinquefasciatus*. Low frequencies (≤3%) of *Cx. quinquefasciatus* alleles for this marker were noted, and mostly confined to populations along the Interstate 25 corridor. Pueblo was distinct in that it had 10% *Cx. quinquefasciatus* alleles, mostly of one allele size. The degree of population genetic structure observed in this study is in contrast with that of *Cx. tarsalis*, the other major vector of WNV in the western U.S., and likely reflects the two species’ different dispersal strategies.

## Introduction

In the new world, the *Culex pipiens* complex of mosquitoes consists of *Cx. pipiens* L., *Cx. quinquefasciatus* Say, their hybrids, and an autogenous form of *Cx. pipiens*, *Cx. pipiens* form molestus Forskål. All taxa are considered efficient vectors of arboviruses such as West Nile virus [1] and St. Louis encephalitis virus [2] in North America. In terms of factors affecting their distribution in the U.S., the most important difference between *Cx. pipiens* and *Cx. quinquefasciatus* is the inability of *Cx. quinquefasciatus* to diapause, which limits its northward spread. The hybrid zone in the U.S. is nevertheless extensive, indicating that a high degree of gene flow has occurred since these species were introduced [3], [4]. For a discussion of theories regarding the introduction of *Cx. pipiens* complex mosquitoes to the New World, see [5].

In this study, we use a combination of microsatellite markers that we recently developed [6] and previously published [7]–[9] microsatellite markers to explore the genetic relationships of a set of populations across a relatively small area, the state of Colorado, USA. Colorado (CO) is bisected by the Rocky Mountains, which reach heights of over 4,000 m and therefore should influence patterns of gene flow among populations of flying insects. The dry eastern plains of the state represent expanses of low-quality habitat that make rivers important conduits for dispersal and colonization by *Cx. pipiens* complex mosquitoes. Also influencing their distribution is a preference for larval sites with organically-enriched stagnant water, which makes these organisms well-adapted to coexist with humans in urban and suburban habitats [10]. Within Colorado, low winter temperatures and high elevations favor *Cx. pipiens*, or specimens that diapause in winter, and pure *Cx. quinquefasciatus* specimens have not been collected in the state. However, ephemeral summertime introductions of *Cx. quinquefasciatus* along transportation corridors likely occur and may result in limited hybridization.

Population genetic studies of disease vectors are useful for their ability to detect and quantify genetic structure in a system. The presence of genetic structure indicates that some barriers to panmixia exist, which can lead to adaptation to local habitats. Such adaptation may play a role in the observed variation in vector competence among populations of *Culex* mosquitoes. For example, Reisen et al. [11] speculated that variation in *Cx. tarsalis* vector competence could be attributable to genetic factors, because other sources of variation had been accounted for. In addition, Kilpatrick et al. [12] found differences in the genetic background of individual *Cx. pipiens* complex mosquitoes that became infected with WNV versus those that did not. Richards et al. [13] asserted that environmental and biological (including genetic) causes of within- and among-population variability in *Cx. quinquefasciatus* vector competence have not been adequately explored and thus could account for such variability. With regard to other mosquito species, Lambrechts et al. [14] determined that vector competence for dengue viruses in *Aedes aegypti* was likely a function of interactions on a genetic level between vector and virus. Bataille et al. [15] argued that habitat differences drive local adaptation in *Ae. taeniorhynchus*, resulting in genetic divergence and site-mediated disease transmission dynamics.

Isolation by distance (IBD) is the tendency for populations to diverge genetically with linear distance. Previous work by our lab on *Cx. pipiens* complex mosquitoes in the central U.S. found a significant IBD effect among the 14 populations sampled, which were separated by an average distance of 112 km [4]. That study, as well as several others in different parts of the U.S. [16]–[18], [5], was on a scale encompassing several states, although work on a similar geographic scale to this study has been done in the state of California [19]. The finding of significant genetic structure at a broad scale suggests that finer scale structure (for example, on the scale of a single state) could elucidate genetic differences among groups thought to be more or less genetically homogenous across populations.

The main objective of this study was to quantify the genetic diversity and differentiation among *Cx. pipiens* populations in Colorado. In addition, this study sought to determine whether the new set of markers, used in combination with previously-published markers, would allow finer scale resolution of genetic differences among populations of closely-related individuals.

## Materials and Methods

### Collection of Specimens

**Figure 1:**
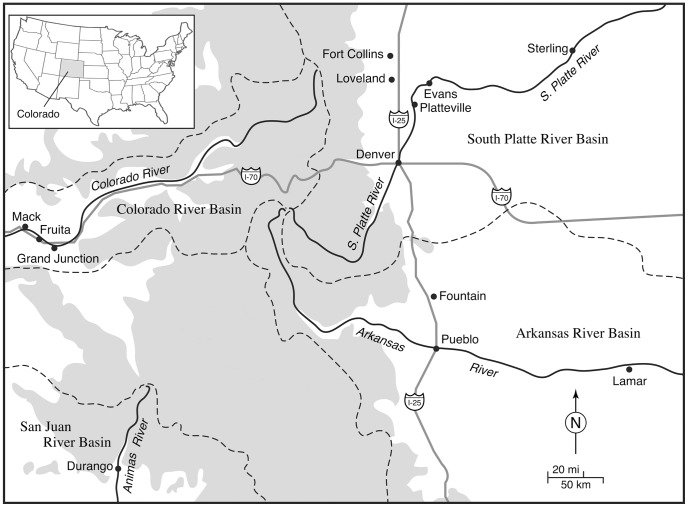
Map of study area. Sample sites (n = 13) are shown, as well as the location of the Rocky Mountains (grey shading), major highways, and relevant waterways. Dashed lines indicate boundaries of the river basins in the state that were sampled in this study.

During July and August 2009, 13 sampling sites were chosen in Colorado, USA among four river basins in the state that are at elevations likely to have suitable habitat for *Cx. pipiens* complex mosquitoes (Figure 1, Table 1). In addition, sites were located on either side of the Rocky Mountains. Elevation among sites ranged from 1107 meters in Lamar, to 1997 meters in Durango (average elevation 1475 meters). Mosquito populations were sampled with one gravid trap [20] and one CDC light trap (J. Hock Co., Gainesville, FL) at each site, and each site was sampled for one or two nights. Gravid traps were set and all specimens were processed in the field from both types of traps as in [4]. Geographic information for sampling sites as well as the numbers of specimens per site (total n = 597) are given in Table 1.

**Table 1:** Site information for specimens in this study.

Site Name	Latitude	Longitude	Elevation (m)	*N*
Durango^a^	37° 16.142′	−107° 52.576′	1997	14
Fruita^b^	39° 13.576′	−108° 52.635′	1372	59
Grand Junction^b^	39° 04.508′	−108° 32.797′	1402	44
Mack^b^	39° 13.344	−108° 52.021′	1378	25
Lamar^c^	38° 04.531′	−102° 37.086′	1107	66
Fountain^c^	38° 40.933′	−104° 42.017′	1690	51
Pueblo^c^	38° 15.700′	−104° 39.395′	1456	67
Denver^d^	39° 42.302′	−104° 58.783′	1609	69
Evans^d^	40° 22.583′	−104° 41.500′	1417	31
Fort Collins^d^	40° 36.489′	−105° 3.877′	1552	57
Loveland^d^	40° 23.867′	−105° 4.450′	1490	36
Platteville^d^	40° 12.750′	−104° 44.667′	1493	23
Sterling^d^	40° 37.314′	−103° 12.941′	1212	55

Elevation of site in meters; N, number of specimens per site. Specimens were collected using either CDC light traps or gravid traps in July-August 2009 in Colorado, USA.

a San Juan River basin,

b Colorado River basin,

c Arkansas River basin,

d South Platte River basin.

### Initial Morphology and Genetic Screening

Specimens were examined using dissecting microscopes and identified to species or lowest taxonomic unit as in [1] using morphological characters in [21]. Overall, collections included approximately the same number of *Cx. tarsalis* individuals as *Cx. pipiens* individuals and the former were not analyzed for this study.

Individual mosquitoes were homogenized in 2 ml tubes using a tissue grinder (Qiagen, Valencia, CA) with a copper BB and 0.5 ml of the diluent BA-1 [1]. DNA was extracted from half of the resulting homogenate, and eluted with 100 ul Buffer AVE (Qiagen). Individuals were initially screened with the ITS PCR assay [22], [23] to confirm membership in the *Cx. pipiens* complex. Only members of the *Cx. pipiens* complex were used in subsequent analyses.

### Microsatellite Analysis

Microsatellite analysis was performed on each individual using a panel of 16 microsatellite loci that was divided into two multiplexes (Table 2). This study uses a new group of loci that were shown to be polymorphic in both *Cx. pipiens* and *Cx. quinquefasciatus*, and effective at discriminating between these two closely-related species [6]. Each reaction consisted of approximately 20 ng of DNA, 1X PCR buffer with Mg, 0.6 mM additional Mg, 200 uM each dNTP (ABS, Foster City, CA), 0.5U hot-start Taq polymerase (Hotstar, Qiagen, Valencia, CA) and primers with the fluorescent labels and in the concentrations listed in Table 2. PCR was carried out in 20 µl volumes in a BioRad DNA Engine PTC-200 thermalcycler (Bio-Rad, Hercules, CA) with the following PCR program: 95° (10 min) to activate the hot-start Taq, 96° (5 min), and 35 cycles of 94° (45 s), 54° (45 s), and 72° (45 s), followed by 72° (10 min). Amplified fragments were visualized with a Beckman-Coulter (Fullerton, CA) CEQ8000 sequencer using the Fragment Analysis Module and a 400-bp size standard. Approximately 10% of the individuals were analyzed twice, and the results from each run were identical.

**Table 2:** Microsatellite loci used or screened for population genetics analyses in this study.

Locus	Dye	Size	*H_E_*	*N*	Multiplex	Conc. (µM)	Source
Cxpq51	D3	162–191	0.645	8	1	0.05	A
Cxpq59	D4	100–118	0.669	7	1	0.02	A
Cxpq68	D4	197–213	0.720	6	1	0.01	A
Cxpq69	D3	280–304	0.330	9	1	0.50	A
Cxpq79	D4	309–337	0.818	6	1	0.08	A
Cxpq109	D2	267–293	0.681	8	2	0.70	A
Cxpq110	D4	186–219	0.573	9	2	0.40	A
Cxpq114	D4	104–119	0.617	6	2	0.05	A
Cxpq117	D3	306–315	0.705	4	2	0.40	A
Cxpq119	D3	212–227	0.679	6	2	0.10	A
CxqGT4F	D4	137–156	0.173	7	2	0.09	B
CxqTri4F	D2	111–126	0.309	6	2	0.04	B
CxpGT46F	D3	259–287	0.832	16	2	0.25	C
CxpGT51F	D2	87–173	0.895	25	1	0.27	C
CxqCTG10	D3	98–116	0.533	6	1	0.05	D
CxqCAG101	D3	184–193	0.605	4	2	0.13	D

Dye, fluorescent label (D2 = black, D3 = green, D4 = blue); Size, range of allele sizes (in bp); H_E_ Expected Heterozygosity averaged across populations; N, number of alleles; Multiplex, which multiplex this locus is part of; Conc., concentration of each primer; Source, original source of locus: A [6], B [8], C [7], D [9].

In addition, specimens were assayed with the locus Cxpq78, which is proposed to be monomorphic in *Cx. pipiens* (fixed at three TCG repeats and 208 bp) and polymorphic in *Cx. quinquefasciatus* (up to nine repeats and range 203–226 bp; [6]). The marker is unsuitable for population genetic analyses of *Cx. pipiens* for this reason, and consequently was not used in the panel of markers. However, Cxpq78 may be useful when looking for signals of genetic admixture between the two species, so allele frequencies of this marker were examined in each population.

### Data Analysis

Allele frequencies were analyzed by the program Microchecker [24] which looks for evidence of null alleles. Multilocus genotypes for each individual were then processed by the program Convert [25] for use in Arlequin [26], Structure [27], and GenePop [28], [29] as well as to produce a table of allele frequencies. Arlequin was used to generate the genetic diversity estimates Observed and Expected Heterozygosity (H_O_ and H_E_) using Nei’s unbiased estimate [30], and this information was used to determine whether there were departures from Hardy-Weinberg equilibrium (HWE) for individual loci within each population using Fisher’s Exact Tests [31]. Arlequin was also used to estimate how genetic variation is partitioned in this system, by conducting several Analyses of Molecular Variance (AMOVA). One included all populations in one group, and two additional analyses examined populations grouped by river basin or grouped by whether they were on the east or west side of the Rocky Mountains. These analyses were later repeated excluding the Lamar population. AMOVAs also generate F-statistics as per Weir and Cockerham [32]. The sample size-corrected method of FSTAT was used to calculate the allelic richness per locus in each population, and FSTAT was also used to look for instances of linkage disequilibrium (LD). To determine whether significant differences existed among the average allelic richness for each population, those values were also compared using a Kruskal-Wallis One-Way ANOVA on Ranks using SigmaStat (Systat Software, San Jose, CA).

Several methods were employed to examine the degree of genetic structure in the study populations. First, Arlequin was used to generate between-population F_ST_ values, with the significance of each value determined by permutation test. The SeqBoot program of Phylip [33] was used to generate 1000 bootstrap replicate data sets of allele frequencies, which were used as input for the Phylip program GenDist, where 1000 replicate distance matrices based on Cavalli–Sforza and Edwards’s chord distance [34] were created. These matrices were used as input for the program Neighbor to generate 1000 neighbor joining trees. The Phylip program Consense was then used to generate a consensus tree with bootstrap support values and the program Drawtree produced an unrooted phenogram of the consensus tree.

Structure was used to determine the most likely number of genetic clusters represented by the data. Ten runs with the default settings (i.e. with correlated allele frequencies and no prior population information) were run for each value of *K* from 1–10 with 50,000 burn-in iterations and 100,000 data collecting steps. The log likelihood values (LnP(D)) were averaged to determine which value of *K* was associated with the highest likelihood value. The program Distruct [35] was used to display the Structure results for the run with the highest posterior probability.

To determine whether there was an isolation by distance (IBD) effect, GENEPOP was used to regress the matrix of linearized F_ST_ values (converted to F_ST_/(1-F_ST_) as per Rousset [36]) on a matrix of log10 distances (in km) between sites. The significance of the comparisons was determined with Mantel’s tests.

## Results

### Genetic Diversity

After Bonferroni correction, there were 35 instances of HW disequilibrium (out of 182 tests) and all instances were due to an excess of homozygotes. Twenty-two of the departures were due to loci Cxpq68 and Cxpq69, so both loci were excluded from further analyses. The remaining 13 instances were distributed among six of the remaining 14 loci, and ranged from 1–4 instances per locus (Table S1). Pueblo, Lamar and Denver had the most instances of departures from HWE (n = 3 each) and other populations with departures included Evans, Fort Collins and Platteville. No significant instances of LD were found between pairs of loci after Bonferroni correction (out of 1014 total comparisons). Microchecker results showed a small percentage of population-locus combinations with evidence of null alleles (9% out of 182 population-loci comparisons) so the dataset was not adjusted for subsequent analyses.

Expected Heterozygosity (H_E_) values were fairly consistent across populations and ranged from 0.542 in Evans to 0.655 in Lamar (Table S1). In all instances, H_O_ < H_E_. The two sites with the lowest elevations, Lamar and Sterling, both located on the eastern side of the state, exhibited the highest H_E_ values. Allelic richness values ranged from 3.77 alleles per locus in Evans to 4.67 in Lamar, but were not significantly different from each other (*P* = 0.45; Table S1).

The results of the Cxpq78 assay indicated that all of the populations on the western side of the mountains were fixed for this locus at 208 bp. In addition, three populations in the northeastern part of the state (Evans, Platteville and Sterling) were also fixed at 208 bp. Several populations (Denver, Fountain, Lamar, Fort Collins, Loveland) had a low percentage (≤3%) of *Cx. quinquefasciatus* alleles and most of these, with the exception of Lamar, were located close to the Interstate 25 (I-25) corridor. Pueblo had the largest percentage (10%) of *Cx. quinquefasciatus* alleles, mostly of one size (226 bp).

### Genetic Differentiation

The overall F_ST_ value was modest but statistically significant (F_ST_ = 0.05, *P*<0.05) indicating detectable genetic structuring in this system. Pairwise F_ST_ values between populations ranged from zero to 0.10 and most comparisons were statistically significant (*P*<0.05; Table S2). Comparisons with the Lamar site produced the highest F_ST_ values. As expected, populations that were proximate tended to have non-significant pair-wise F_ST_ comparisons (Table S2). The AMOVA that treated all populations as belonging to one group found most variation exists within individuals (85.27%), followed by that attributed to among individuals within populations (10.11%) and among populations (4.61%). The AMOVAs that included an additional level of organization indicated that small but statistically significant (*P*<0.05) amounts of genetic variation are attributable to grouping the populations by river basin (F_CT_ = 0.03; Table 3) or by their position relative to the mountains (F_CT_ = 0.03; Table 4). The variance partitions for the AMOVAs without the Lamar population were still significantly different from zero, and resulted in a lower F_ST_ value (0.04) but the same F_CT_ values (0.03 for grouping populations either by river basin or by side of the mountains).

**Table 3:** Analysis of Molecular Variance (AMOVA) results when populations are grouped by river basin.

Source of variation	d.f.	Sum of Squares	Variance Components	Percentage of Variation
Among groups	3	118.369	0.100	2.52
Among populations within groups	9	135.351	0.116	2.93
Among individuals within populations	584	2421.137	0.402	10.14
Within individuals	597	1995.500	3.343	84.41
Total	1193	4670.356	3.953	

The amount of variation in each partition was significantly different from zero (*P*<0.05) via permutation test. Corresponding fixation indices are given in the text.

**Table 4:** Analysis of Molecular Variance (AMOVA) results when populations are grouped by East or West side of Rocky Mountains.

Source of variation	d.f.	Sum of Squares	Variance Components	Percentage of Variation
Among groups	1	60.231	0.098	2.44
Among populations within groups	11	193.488	0.149	3.72
Among individuals within populations	584	2421.137	0.402	10.06
Within individuals	597	1995.500	3.343	83.77
Total	1193	4670.356	3.990	

The amount of variation in each partition was significantly different from zero (*P*<0.05) via permutation test. Corresponding fixation indices are given in the text.

**Figure 2:**
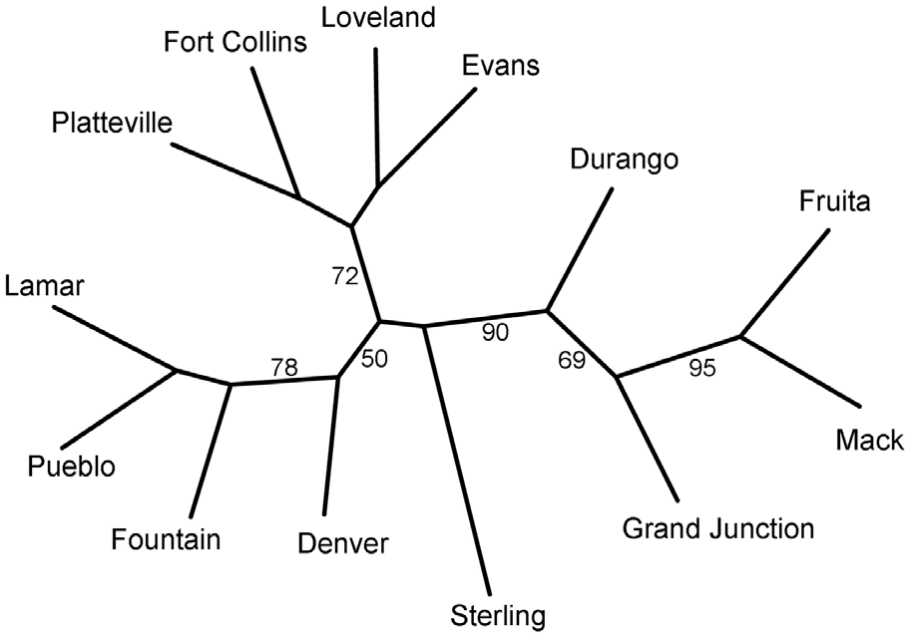
Unrooted neighbor joining consensus tree of thirteen populations estimated from Cavalli-Sforza and Edwards chord distances. Values at the nodes indicate percentage bootstrap support after 1000 replicates.

**Figure 3:**
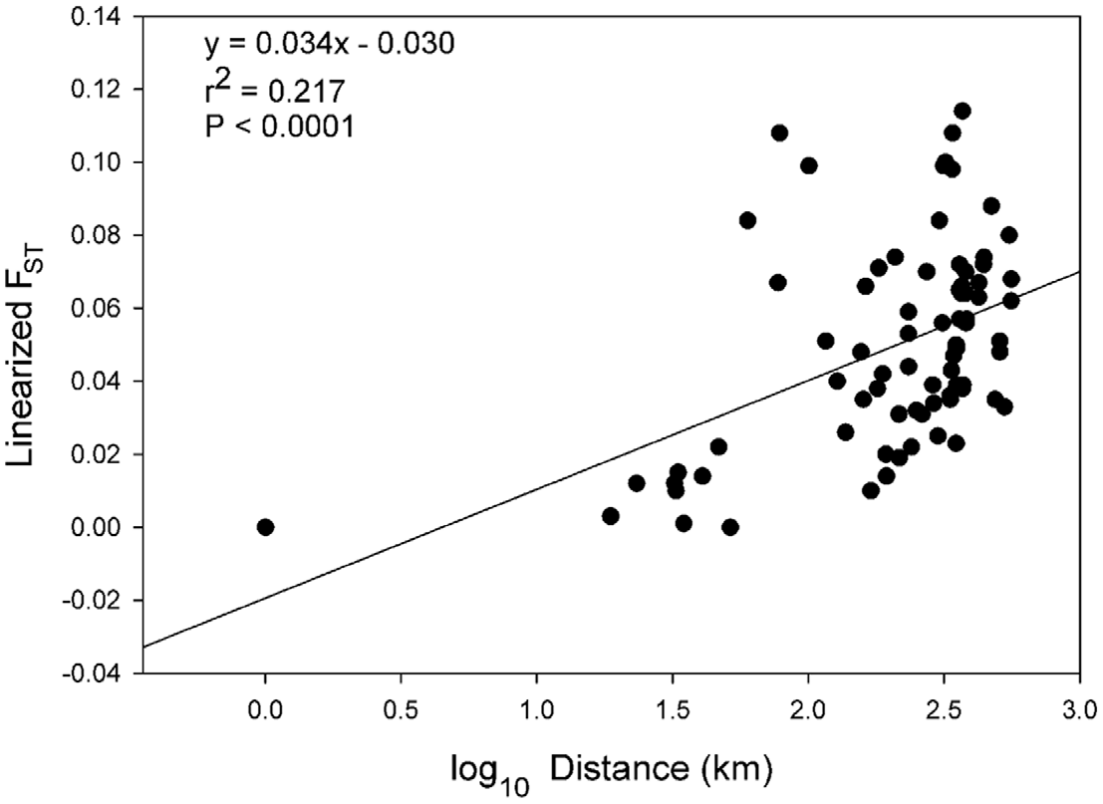
Graph of isolation by distance (IBD) results. Linearized F_ST_ values (F_ST_/(1-F_ST_) are regressed upon the log_10_ linear distance (in km) between pairs of populations, showing a significant IBD effect (Mantel’s test *P*<0.0001; r^2^ = 0.21).

The neighbor joining tree based on Cavalli-Sforza and Edwards chord distances is consistent with the geographic distribution of the populations (Figure 2). The four populations on the west side of the mountains form a group with high bootstrap support (90% of trees). The three populations in the Colorado River basin grouped together (69%), and the Durango population, in the San Juan River basin, was somewhat differentiated. Also showing limited bootstrap support was the group of Platteville, Fort Collins, Loveland and Evans (72%), which are in the South Platte River basin. Sterling and Denver are in the same river basin and the branches for these populations were located close to the one for the Platteville-Fort Collins-Loveland-Evans group, although these branches had somewhat less bootstrap support. The populations in the Arkansas River basin, Lamar, Pueblo and Fountain grouped together with moderate support (78%). There was a significant IBD effect (Mantel’s test *P*<0.0001; r^2^ = 0.21) indicating a positive relationship between genetic differentiation and linear distance (Figure 3). The data point representing the comparison of Mack and Fruita, populations located about 1 km apart, was at the origin of the plot, while data points representing comparisons between other pairs of proximate populations occupied a cluster of points in the lower-center portion of the graph. The remaining points were in a third grouping in the upper right portion of the plot.

### Cluster Analyses

**Figure 4:**
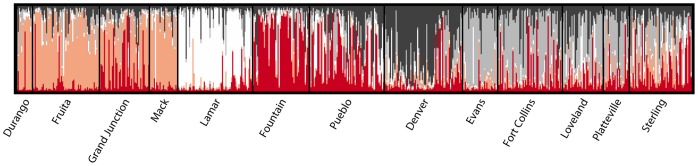
Structure diagram showing most likely number of clusters, *K* = 5. Cluster membership was as follows: Cluster 1- Durango, Fruita, Grand Junction, Mack; Cluster 2– Lamar; Cluster 3– Fountain; Cluster 4– Denver; Cluster 5– Evans, Fort Collins, Loveland, Platteville. Pueblo and Sterling appear highly admixed. The program was run with the default parameters, (i.e. excluding population information) using a panel of 14 microsatellites.

Using the approach outlined in Structure’s documentation, we examined log likelihood and alpha values, as well as the degree of variation among successive runs at the same *K* and determined that Structure grouped the specimens in this study into five clusters (Figure 4). Denver, Lamar and Fountain were clearly each in their own cluster. In addition, Durango, Fruita, Grand Junction and Mack, located on the west side of the mountains, comprised a fourth cluster, and Evans, Fort Collins, Loveland and Platteville formed a fifth distinguishable cluster. The remaining two populations, Pueblo and Sterling, were relatively admixed by comparison and did not form their own clusters. However, the Pueblo population was somewhat similar to the geographically close Fountain population, while the Sterling population was more heterogeneous in composition.

## Discussion

Our results indicate that genetic diversity is fairly consistent across populations, with no sites or groups of sites showing strikingly larger or smaller values. This is perhaps due in part to the scale at which populations were sampled, which is smaller than similar studies of *Cx. pipiens* complex populations in the U.S. The highest H_E_ values came from the two easternmost populations in the study (Lamar and Sterling), which are the two lowest-elevation sites and located on the eastern plains. This observation suggests there may be environmental factors that reduce genetic diversity of *Cx. pipiens* complex populations. For example, lower elevation sites may experience comparatively fewer climatic extremes, which could allow for a greater variety of successful genotypes in those areas. Conversely, it could be that weather conditions at the higher elevation sites reduce a greater percentage of diapausing populations compared to lower elevation sites, resulting in populations with moderately lower genetic diversity.

It is interesting to note that all of the differences between observed and expected heterozygosity within populations in this study were such that H_O_ < H_E_, indicating an excess of homozygotes. In several populations, including Lamar, Pueblo, Denver, Evans, Fort Collins and Platteville, several of the differences were statistically significant and resulted in departures from HWE. One possible explanation for the observed homozygote excess is the Wahlund Effect [37], whereby differences in allele frequencies due to drift among structured subpopulations (here referred to as populations) result in an overall deficit of heterozygotes. When subpopulations are considered as a population, divergent allele frequencies cause fewer heterozygotes overall than would be expected.

The detection of *Cx. quinquefasciatus* alleles at the Cxpq78 locus in some of the study populations suggests that Colorado is a site of limited gene flow between the two species in the *Cx. pipiens* complex. The presence of *Cx. quinquefasciatus* alleles at low frequencies in populations close to the I-25 corridor offers a possible mechanism for their movement, which may occur by anthropogenic means. A similar scenario was proposed for the movement of *Aedes aegyptii* in the state of Arizona, USA, because local conditions made observed dispersal patterns unlikely by natural means [38]. The population with the highest frequency of *Cx. quinquefasciatus* alleles, Pueblo, has some of the warmest temperatures in the state [39]. Notably, there were no *Cx. quinquefasciatus* alleles detected in any of the populations west of the Rocky Mountains. This may be due to high elevation areas and dry conditions of western Colorado, which would impede dispersal through those areas. In addition, the road system on the west side of the mountains is such that there is no major north-south highway through the area, reducing chances of human transport.

Our study has shown that *Cx. pipiens* populations in Colorado are not panmictic. For example, most of the pairwise F_ST_ comparisons were significant (Table 4), except for populations located in close proximity to each other. Also, there was a significant IBD effect, indicating that genetic differentiation increases with distance. The pattern of data points shown in Figure 3 is likely related to our sampling methods, because the two groups of points in the lower part of the plot are from pairs of populations located comparatively close to each other. Data points from pairs of populations that are further apart generally share significant pairwise F_ST_ values and are located in the upper right portion of the plot. The AMOVA analyses showed that moderate but statistically significant amounts of genetic variation can be attributed to grouping populations either by their position relative to the mountains or by river basin. These findings are consistent with the results of the analysis of genetic distances that produced the neighbor joining tree (Figure 2). The tree shows particularly good support for grouping populations on either side of the mountains, as well as the groups of populations in north-central and southeast Colorado, and is generally in agreement with the geographic locations of populations in this study. Sterling was an exception and was located between the western sites and other eastern sites, perhaps due to it experiencing more gene flow from migration than other eastern populations, as suggested by the Structure results.

Although many of the larger differences among sites are between populations on either side of the mountains, comparisons with the Lamar population produced the largest pairwise F_ST_ values. Taken with its higher genetic diversity, low elevation and because it occupies its own cluster in Structure, Lamar appears different from the other CO populations. Whereas Denver, which also occupied its own cluster in Structure and had relatively high genetic diversity, receives a high volume of vehicle traffic and represents a large area of suitable habitat, Lamar is smaller and comparatively remote. These features could account for Lamar’s genetic characteristics, despite its location near a potential dispersal corridor in the Arkansas River. In order to determine whether the genetic differentiation observed in Lamar disproportionately influenced the results of the AMOVAs, analyses were performed excluding this population. The fixation indices were still statistically significant, but their values were smaller, indicating that while Lamar is differentiated from the rest of the populations, it does not account for all of the structure in this system. The neighbor joining tree is consistent with this finding, in that Lamar is located on one end of the tree, but does not have a particularly long branch length.

Despite evidence of statistically significant amounts of genetic differentiation, our results indicate some amount of gene flow among all collections. The mechanism for facilitating the movement of *Cx. pipiens* mosquitoes around the significant physiographic barriers present in the state is unknown at this time, although other studies have suggested anthropogenic or weather-induced movement, as well as dispersal routes around the mountains [40], [38], [41]–[43]. The frequency of significant pairwise F_ST_ comparisons in the current study is in contrast to findings of a study of the congener *Cx. tarsalis*, which did not find significant differentiation among populations sampled from a smaller, five county area in north-central Colorado [41]. The difference could be due to life history characteristics of the two species, as *Cx. tarsalis* is thought to be routinely capable of dispersing long distances, and thrives in habitats such as those provided by agricultural irrigation.

The Structure results indicated that *K* = 5, and were obtained with the parameters set such that the program used only genetic information, not each individual’s population of origin. This finding is in contrast with other studies of the *Cx. pipiens* complex across larger areas [16]–[18], [4], [44] that discerned taxon-level, but not population-level genetic structure. We propose that our findings are the result of the additional microsatellite markers we used, which have allowed finer-scale resolution of genetic relationships among this group of closely related organisms.

Recent studies on *Culex* mosquitoes in Colorado deployed CDC light traps and focused on the congener *Cx. tarsalis*, which is also an efficient vector of West Nile virus in the western U.S. [45], [46], [41], [47]. Among findings of the above studies was a positive association between river corridors and the transmission of West Nile virus, and a greater abundance of *Culex* mosquitoes at lower elevations (1200–1600 m). Epidemiological work conducted on WNV in Colorado has included mapping the locations of disease cases [48]. For example, Figure S1 shows the occurrence of WNV neuroinvasive disease cases by county in Colorado from 2003–2010. When this map is examined with Figure 1, it is apparent that more cases of WNV occur in the lower elevations than in the mountains [41], [48]. For example, the area around Grand Junction is lower in elevation than surrounding counties. In contrast, areas of high elevation, corresponding to the gray shading in Figure 1, have comparatively fewer cases. Although many of these cases are assumed to be infected by *Cx. tarsalis*, most of the shaded counties in Figure S1 (e.g. Larimer, Boulder, Mesa, Pueblo) have urban areas that possess suitable habitat for *Cx. pipiens* complex mosquitoes [49], [50], and we collected about equal numbers of both species at our collection sites. Future disease modeling efforts that include data on both primary vectors, *Cx. tarsalis* and *Cx. pipiens*, will prove more realistic.

Since dispersal and demographic changes are difficult to track in small organisms such as mosquito vectors, population genetics analyses can be employed to inform researchers and disease modelers about these epidemiologically important characteristics. In our study, we performed such analyses for *Cx. pipiens* populations in Colorado, and suggest our work as an example of how genetic data can complement epidemiological data in characterizing a disease vector. Future studies that incorporate population genetics, and epidemiological data on human disease will lead to comprehensive models of disease transmission.

## Supporting Information

Figure S1 Incidence map of West Nile virus neuroinvasive disease in Colorado, USA 2003–2010. Map shows collection sites and West Nile virus neuroinvasive incidence rates by county.
(TIF)

Table S1 Genetic diversity values for 14 Colorado *Culex pipiens* complex populations in this study.
(XLSX)

Table S2 Pairwise F_ST_ values between pairs of *Culex pipiens* complex populations in Colorado.
(XLSX)
