# Supplementary figures and images for: Characterization of Culex pipiens Complex (Diptera: Culicidae) Populations in Colorado, USA Using Microsatellites
Source: PLoS One. 2012 Oct 19;7(10):e47602. doi: 10.1371/journal.pone.0047602 (PMC3477124; doi:10.1371/journal.pone.0047602)

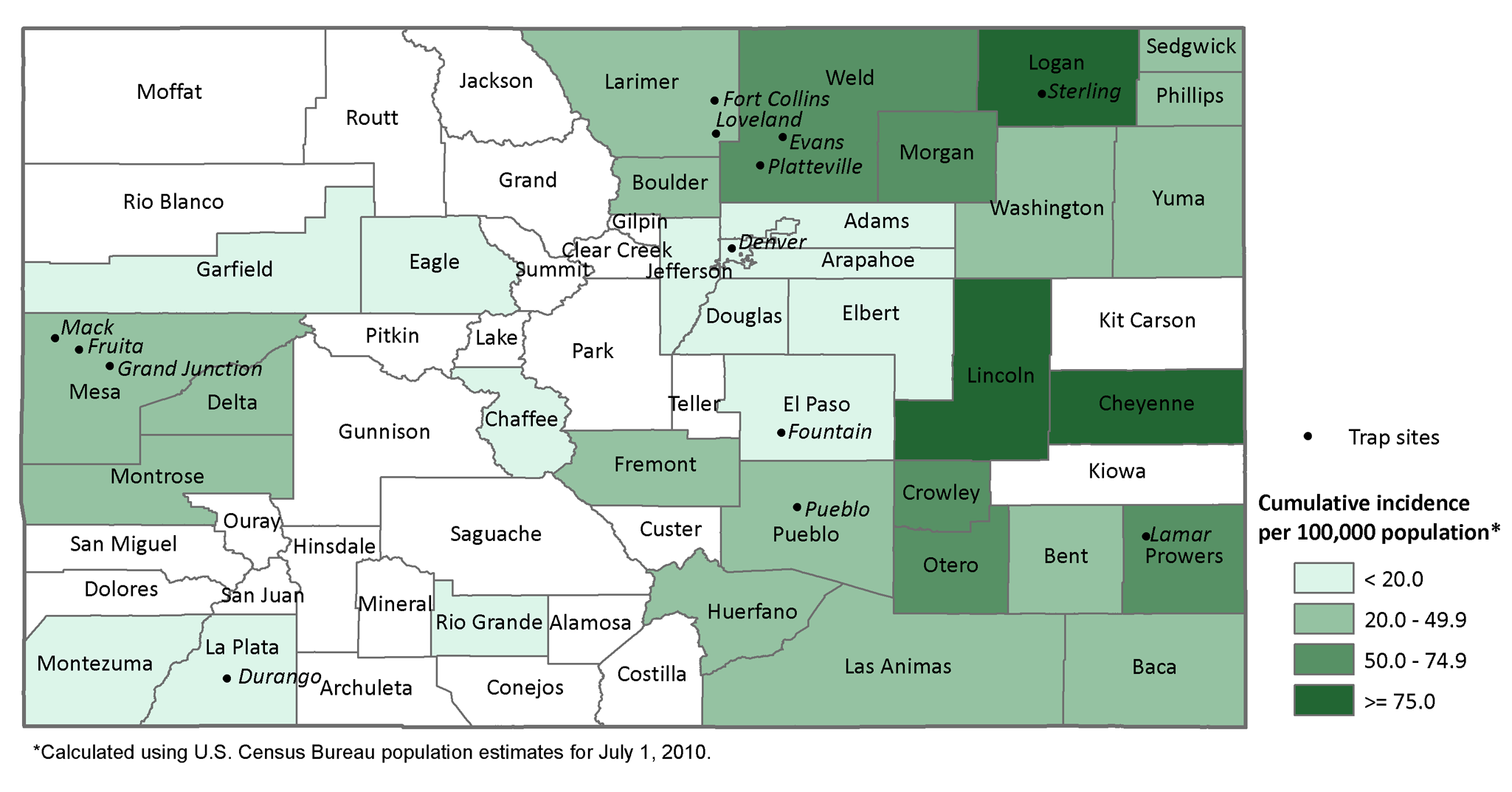

Supplement: Figure S1 — Incidence map of West Nile virus neuroinvasive disease in Colorado, USA 2003–2010. Map shows collection sites and West Nile virus neuroinvasive incidence rates by county. (TIF) [file pone.0047602.s001.tif]
